# Comparative analysis of the health status of the population in six health zones in South Kivu: a cross-sectional population study using the WHODAS

**DOI:** 10.1186/s13031-021-00387-0

**Published:** 2021-07-02

**Authors:** Samuel Lwamushi Makali, Espoir Bwenge Malembaka, Anne-Sophie Lambert, Hermès Bimana Karemere, Christian Molima Eboma, Albert Tambwe Mwembo, Steven Barnes Ssali, Ghislain Bisimwa Balaluka, Phillippe Donnen, Jean Macq

**Affiliations:** 1 Ecole Régionale de Santé Publique, Faculté de Médecine, Université Catholique de Bukavu, Bukavu, Democratic Republic of Congo; 2 Hôpital Provincial Général de Référence de Bukavu (HPGRB), Bukavu, Democratic Republic of Congo; 3 Institut de Recherche Santé et Société, Université Catholique de Louvain, Brussels, Belgium; 4 Ecole de Santé Publique, Université de Lubumbashi, Lubumbashi, Democratic Republic of Congo; 5 Makerere University, Kampala, Uganda; 6 Ecole de Santé Publique, Université Libre de Bruxelles, Brussels, Belgium

**Keywords:** Health status, Population, WHODAS, Conflict, South Kivu, DRC

## Abstract

**Background:**

The eastern Democratic Republic of Congo (DRC) has experienced decades-long armed conflicts which have had a negative impact on population’s health. Most research in public health explores measures that focus on a specific health problem rather than overall population health status. The aim of this study was to assess the health status of the population and its predictors in conflict settings of South Kivu province, using the World Health Organization Disability Assessment Schedule (WHODAS).

**Methods:**

Between May and June 2019, we conducted a community-based cross-sectional survey among 1440 adults in six health zones (HZ), classified according to their level of armed conflict intensity and chronicity in four types (accessible and stable, remote and stable, intermediate and unstable). The data were collected by a questionnaire including socio-demographic data and the WHODAS 2.0 tool with 12 items. The main variable of the study was the WHODAS summary score measuring individual’s health status and synthesize in six domains of disability (household, cognitive, mobility, self-care, social and society). Univariate analysis, correlation and comparison tests as well as hierarchical multiple linear regression were performed.

**Results:**

The median WHODAS score in the accessible and stable (AS), remote and stable (RS), intermediate (I) and unstable (U) HZ was 6.3 (0–28.6); 25 (6.3–41.7); 22.9 (12.5–33.3) and 39.6 (22.9–54.2), respectively. Four of the six WHODAS domain scores (household, cognitive, mobility and society) were the most altered in the UHZs. The RSHZ and IHZ had statistically comparable global WHODAS scores. The stable HZs (accessible and remote) had statistically lower scores than the UHZ on all items. In regression analysis, the factors significantly associated with an overall poor health status (or higher WHODAS score) were advanced age, being woman, being membership of an association; being divorced, separated or widower and living in an unstable HZ.

**Conclusions:**

Armed conflicts have a significantly negative impact on people’s perceived health, particularly in crisis health zones. In this area, we must accentuate actions aiming to strengthen people’s psychosocial well-being.

## Background

Regions affected by armed conflict are far behind in achieving their development goals [1]. Beyond death and physical trauma directly attributable to armed conflict, the resulting socio-political instability leads to a discontinuation of the provision of health services, the limitation of access to care and a dysregulation of health system governance [2–4]. Conflicts also has an impact on the socio-economic life of people, the education of citizens and contributes to the impoverishment of communities [5–9].

All these factors create conditions that interfere with strategies for control and response to communicable diseases. Protracted conflict is also associated with food insecurity and high maternal and child morbidity and mortality [3], and ultimately affects the health status of the general population [10, 11].

The Democratic Republic of Congo (DRC) has been at the heart of repeated armed conflicts for several years. The main conflicts, including the 1996’s described as “world war for Africa” [12], took place mainly in the eastern part of the country (particularly in the provinces of Ituri, North Kivu and South Kivu). As a result of these conflicts, the national crude death rate rose from 1.3 deaths per 1000 population per month in 1997 to 2.2 deaths per 1000 population per month in 2002 [13]. This rate would exceed by 40% (for the whole country) and 60% (in the East of the country) the African average in 2013 [14].

Since 1993, an estimated 4.1 million people have been internally displaced in the east of the country [15]. At the same time, there has been a proliferation of armed groups in this region. According to the Human Right Watch December 2018 report, more than 140 armed groups are active in North and South Kivu [16].

Armed conflicts have weakened the health system, affecting both health personnel and health facilities. For example, there have been a total of 96 incidents targeting the health system in South Kivu between April 2017 and October 2019 [17]. Another striking fact is the outbreak of armed conflicts in Bijombo (Uvira, South Kivu) which has resulted in nearly 3500 internally displaced persons (IDPs). This events also provoked the closure of the general referral hospital and eight other health facilities, depriving nearly 60,000 people of health care in 2018 [18].

Some authors assumed that humanitarian aid, focused on vertical programs, contributes to the effective performance of the health system in terms of health care provision [19]. In South Kivu, at the end of January 2018, there were almost 36 humanitarian actors working on 51 ongoing projects [20]. Nevertheless, several indicators of population health remain generally worrying in South Kivu. The South Kivu province has one of the highest prevalence of stunting in children under 5 years old (53%) [21] and the highest infant mortality rate in the country (92‰) [22].

In DRC, the health status of a population is often assessed using health indicators that reflect disease-related mortality and/or morbidity (child mortality, malaria-related morbidity, …) [23]. This way of measuring health status does not consider other aspects of the person (social, psychological) that seem to have a significant impact on that person’s health, especially in crisis situations related to armed conflict.

Despite the growing recognition in the global literature of the deleterious effects of armed conflict on health systems, few studies have assessed health status at the community level in the provinces of Kivu. Available studies, which used World Health Organization Disability Assessment Schedule (WHODAS), have focused on specific populations [24], without rigorously assessing the real impact of crises linked to armed conflict on communities’ health. Also, in most studies on the health status of a population, the focus is usually on measures focused on deaths or specific health problems.

This study assessed the health status of people in health zones of South Kivu with different socio-political and security profiles. We used the World Health Organization Disability Assessment Schedule (WHODAS) to measure the health status of the population and its associated factors.

## Methods

### Study design and setting

We conducted a community-based cross-sectional and household survey from May to June 2019.

This study was carried out in South Kivu, one of the eastern provinces of DR Congo. This province has one capital city (Bukavu) and eight territories. Its population was estimated at 6,937,726 inhabitants in 2018 (three quarters of whom live in rural areas), distributed over 64,791Km^2^ [25]. In terms of healthcare services governance, the province has two levels: the provincial health division is composed of several health programs, and supports the health zone. Each health zone has a network of primary and secondary level health facilities centered around a referral hospital [14]. Among the 34 health zones (HZs) of the South-Kivu provinces, six were selected, of which five were rural (Mulungu, Fizi, Walungu, Bunyakiri and Idjwi) and one urban (Kadutu). The selection of these health zones was made arbitrarily, considering their socio-demographic differences and security settings.

The Kadutu health zone is a mixed zone (urban and peri-urban) without large-scale armed conflicts for more than 5 years. Idjwi Island is a rural health zone that has been protected from the direct effects of armed conflict in eastern DR Congo, due to its geographic location. The other four health zones (Bunyakiri, Walungu, Mulungu et Fizi) are rural and have different profiles regarding armed conflict and international aid.

### Typology of health zones

The armed conflict level was defined for each health zone by using two parameters: the number of deaths directly related to armed conflict (BRD) and the number of internally displaced persons (IDPs).

For the first parameter, we used the raw data on deaths attributable to armed conflict. This was extracted from the Uppsala Conflict Data Program Battle-related Deaths Dataset (UCDP BRD) database from 2013 to 2018 [26–28]. This database has several variables including the type of conflict, the location of the conflict (up to the level of territories of a province) as well as the geolocation data of the conflict from 1989 to 2018. Created for research purposes, UCDP BRD defined deaths related to armed conflict as “deaths caused by warring parties that may be directly related to battle” [28]. This data base seemed to be more complete for our study than others such as the ACLED (Armed Conflict Location Events Dataset) [29, 30]. Health zones mapping data was obtained through shapefile downloaded from the United Nations Office for the Coordination of Humanitarian Affairs (UNOCHA) database [31]. We then merged the health zones mapping and BRD data with Quantum Geographic Information System (QGIS) software which allowed us to put each BRD in its health zone. The BRD was obtained by dividing the total number of deaths related to armed conflicts from 2013 to 2018 by the average of the population from 2013 to 2018. Thus, the BRDs were 2.4, 51.7, 0.4, 0, 21 and 0.4 (per 100,000 inhabitants) respectively for the Bunyakiri, Fizi, Idjwi, Kadutu, Mulungu and Walungu health zones. A health zone which more than 5 BRD per 100,000 inhabitants was considered to be in conflict [32].

For the second parameter, we used UNOCHA data on IDPs for the South Kivu territories in which the health zones were located. The data available were those for 2014 and 2017 [33, 34]. We used the 1998 OCHA guideline to define IDPs [35]. Thus, the number of IDPs during the 2 years was 434,014; 244,798; 6600; 2755; 16,7010 and 47,965 respectively in the territories of Kalehe (Bunyakiri HZ), Fizi (Fizi HZ), Idjwi (Idjwi HZ), City of Bukavu (Kadutu HZ), Shabunda (Mulungu HZ) and Walungu (Walungu HZ). An arbitrary threshold of over 50,000 IDPs was considered suggestive of a conflict heath zone based on the average number of IDPs recorded during 2017 in the six territories, considering that the DRC recorded the highest number of IDPs in Africa that year [34].

Considering the two parameters, and referring to other studies [11, 32], there were three possibilities defining the three types of health zones: (1) health zones that had less than 5 BRD from 2013 to 2018 and who registered less than 50,000 IDPs during the years 2014 and 2017. These health zones (Kadutu, Idjwi and Walungu) were called “stable”. This class was split into two groups to highlight the feature of the Idjwi heath zone. Kadutu and Walungu health zones were ultimately considered to be “accessible and stable” (AS) and Idjwi as “remote and stable” (RS); (2) those with more than 5 BRDs and a high number of IPDs (more than 50,000) were considered as “unstable”. Mulungu and Fizi fulfilled this condition; (3) finally, health zones that had one of the parameters high and the other low.

according to the thresholds established (Bunyakiri) were considered as “intermediate”.

### Study size

Using the Stata 15 sample calculation package for analysis of variance, considering an intergroup variance of 393 as observed in a recent study in South Kivu [24], and the mean values of WHODAS of 8.2, 10.2 and 12.2 for the conflicting, intermediate and stable areas respectively, and assuming a type 1 error of 5% with a power of 80%, the minimum sample size required was 1425 participants. And so, we collected data from 48 people per village, or 240 people per health zone, which gave us a total of 1440 participants.

### Selection of participants and sampling procedure

The population of this study was made up of people aged 18 or over, residing in the health zone for more than 1 year. Multi-level sampling was carried out. At the first stage, five health areas were randomly selected from each health zone. Within each health area, 48 households were then randomly selected. Three of the selected health areas (two in Mulungu and one in Bunyakiri) were not accessible due to accessibility and insecurity issues. They were replaced by three neighboring health areas which were more accessible. In each health area, the list of households was obtained from the village chief. From a “starting point” point, we choose the direction (using direction indicated by the tip of a discarded pen) to select the first household and the rest were selected as the closest one. Within each household, the adult who was found there during a first visit was interviewed. Investigators visited homes twice to capture those who were not present (for many reasons) during the day.

Excluded were all those with confirmed psychiatric disorder who were not able to provide an informed consent.

### Data collection

A structured questionnaire was used to collect socio-demographic and economic data from the participants. WHODAS, originally in English, has been translated into Kiswahili and French by the team of linguists from the School of Languages at the Catholic University of Bukavu, following the WHO principle of translation and back-translation. The pre-test of the questionnaire was carried out with 30 people (15 with French and 15 with Swahili questionnaire) living in the same community where our study was conducted. The administration of the questionnaire took between 15 and 20 min. The adaptations were integrated into the final instrument. Data collection was done by six doctors trained in observing ethical principles, confidentiality and non-maleficence, and also on the correct use of WHODAS.

### Variables, instrument and measurements

The main variable of the study is the WHODAS summary score measuring an individual’s health status. The WHODAS score ranges from 0 to 48 (each of 12 items rated from 0 to 4), measuring the cognitive, functional and social performance of the participants; the lower the score the better the health status.

The WHODAS (with 12 items) is a standardized WHO questionnaire with good psychometric properties [36, 37], adapted to several cultures and validated in several countries [38], including among children in rural areas of middle and low income countries [39]. In a recent study, it was shown that WHODAS can be used to measure the health status of populations in rural and semi-urban areas of South Kivu [24].

We converted this score into a new score ranging from 0 to 100 to make the comparison and interpretation easy. Even if there is no agreed for cut-point identifying persons with significant disability, as says by Andrews et al. (40)0), a person who has a WHODAS score above 10 out of 48 (21 out of 100 in our study) is likely to have clinically significant disability. The other levels of disability according to the score achieved were: 0 (presumed to be without disability, 2–9 (with mild disability), 10–20 (with moderate disability).

We don’t perform any factor analysis in our study to transform the WHODAS items. But to better understand which components of daily life of people living in areas of chronic crisis related to armed conflict are most impaired compared to people living in stable areas, we used the results of the exploratory principal components analysis of Andrews on the general population in Australia [40]. The 12 items WHODAS were condensed into 6 domains of disability (assisting and completing daily tasks, acquiring and using information, moving and handling objects, taking care of self, interacting with others and participating in society). Each domain was scored out of 8 because it represented the sum of two WHODAS items (each normally scored out of 4).

The socio-cultural and demographic characteristics as well as the type of the health zone of residence were analyzed as explanatory variables. We defined the socioeconomic status of the respondents according to the procedure developed by Filmer and Pritchett [41] based on the possession of durable household assets and used in demographic and health surveys (for example computer, mobile phone, radio, permanent electricity, …). The socio-demographic status was therefore obtained thanks to a multiple correspondence analysis grouping together 14 sub-variables and thus defining three classes (low, medium and high). The variable « membership of a local saving association » brought together people who were part of any development association in the community.

### Data management and statistical methods

The data was encoded in Epi Info 7 and exported to Excel for cleaning. The analysis was performed with IBM SPSS version 25 software. The frequencies with proportions and medians with interquartile ranges were used respectively to summarize respectively nominal and discrete or continuous variables. We used the Box-Cox transformation to normalize our dependent variable, the WHODAS score. Chi-square test was used to compare categorical variables while nonparametric tests (Kruskall-Wallis and Wilcoxon with Bonferroni adjustment) were used to compare discrete or continuous variables, including WHODAS score. The Z-test, with Bonferroni adjustment, was used to compare the characteristics of the population and the types of health zones. A hierarchical linear regression was performed to examine the contribution of each factor to the explanation of the variance of the WHODAS score. We test two models: the first representing only the socio-demographic characteristics of the participants and the second incorporating the level of crisis (or the type of health zone) into the first model. There was no multicollinearity problem because all of the variance inflation factor (VIF) values were < 1.5. Only the variables having a *p*-value < 0.05 in the analyses of variance with one factor (ANOVA with 1 factor) or of correlation test of Pearson were introduced in the models. We finally choose the model which has a lower AIC (Akaike information criteria).

### Ethical considerations

To carry out this study, we had the approval of the Ethics Committee of the Université Catholique de Bukavu. A letter from South Kivu’s head of health division, who gave his approval for the study, was presented to the health authorities in the health zones visited. Only people who gave prior informed consent participated in this survey.

## Results

### Characteristics of the population and their distribution in types of health zones

These characteristics are presented in Table 1. The median age and the gender distribution of respondents were identical in all types of health zones. The Shi and Havu tribe were more common in stable health zones. The proportion of Catholics and formal employees was higher in AS health zones. The major occupation in other health zones was conducting small business as compared to the AS health zone. Temporary housing and medium socioeconomic level characterize the population living in UHZs. Development associations were more common in the RS HZ.

**Table 1:** Sociocultural and demographic characteristics and univariate analysis by types of health zones (*n* = 1440)

Variables	Accessible and stable (AS)	Remote and stable (RS)	Intermediate (I)	Unstable (U)	p(*)(**)
**Age (year)**	38 (25–52)	32 (25–42)	31 (23–46)	37 (26–53)	*p(I/LE) = 1 et p (RS/C) = 1*
**Sex**					
Male	195 (40.7)	100 (42.0)	92 (38.3)	211 (44)	*p = 0.5 2 décimales*?
Female	284 (59.3)	138 (5.08)	148 (61.7)	269 (56)	*P = 0.5*
**Marital statues**					
Never married	112 (23.4)	33 (13.8)	39 (16.4)	96 (20)	*p (RS/LE) = 0.01*
Married	308 (64.4)	174 (72.5)	162 (68.1)	308 (64.2)	*p > 0.05*
Separated or divorced	8 (1,7)	7 (2.9)	17 (7.1)	29 (6)	*p(I;C/RS) = 0.001/0.003*
Widower	50 (10.5)	26 (10.8)	20 (8.4)	47 (9.8)	*p > 0.05*
**Tribe**					
Shi and Havu	396 (82.7)	155 (65.1)	27 (11.3)	6 (1.3)	*p (RS;LE/C;I) = < 0.001*
Rega	27 (5.6)	1 (0.4)	35 (14.6)	236 (49.5)	*p(C;I/RS;LE) = < 0.001*
Bembe	0 (0)	0 (0)	0 (0)	114 (23.9)	/
Tembo	1 (0.2)	0 (0)	58 (24.3)	0 (0)	*p(I/RS) = < 0.001*
Others	55 (11.5)	121 (25.4)	119 (49.8)	121 (25.4)	*p(I/RS;LE;C) = < 0.001*
**Religion**					
Catholic	306 (63.7)	81 (34)	73 (30.4)	161 (33.6)	*p (RS/LE;I;C) = < 0.001*
Protestant	158 (32.9)	141 (59.8)	123 (51.2)	253 (52.8)	*p (LE;I;C/RS) = < 0.001*
Muslim	10 (2.1)	0 (0)	7 (2.9)	43 (9)	*p(C/RS;I) = < 0.001/0.008*
Others	6 (1.3)	16 (6.7)	37 (15.4)	22 (4.6)	*p (LE;I;C/RS) = < 0.001and p(I/LE;C) = < 0.001/0.15*
**Respondent’s occupation**					
Formal employee	76 (15.9)	11 (4.6)	23 (9.6)	35 (7.3)	*p (RS/LE;C) = < 0.001*
Occasional work	0 (0)	0 (0)	1 (0.4)	18 (3.8)	*p(C/I) = 0.009*
Small business	104 (21.7)	131 (54.8)	126 (52.7)	278 (58)	*p (LE;I;C/RS) = < 0.001*
Farmer	54 (11.3)	40 (16.7)	17 (7.1)	51 (10.6)	*p (LE/I) = 0.007*
Unemployed	245 (51.1)	57 (23.8)	72 (30.1)	97 (20.3)	*p (SA/LE;I;C) = < 0.001 and p(I/C) = 0.02*
**Number of adults in the household**	4 (2–5)	2 (2–3)	3 (2–4)	3 (2–4)	*P (SE/C) = 0.29 and p(C/I) = 0.42*
**Type of housing**					
Temporary	232 (48.3)	112 (46.9)	125 (52.1)	294 (61.4)	*P(C/RS;LE) = < 0.001/0.001*
Semi-permanent	197 (42)	79 (33.1)	91 (37.9)	122 (25.5)	*P (RS;I/C) = < 0.001/0.003*
Permanent	51 (10.6)	48 (20.1)	24 (10)	63 (13.2)	*P (LE/RS;I) = 0.003/0.012*
**Number of children < 5 years old in the household**	1 (0–2)	1 (0–2)	2 (1–3)	2 (1–3)	*P (RS/LE) = 1 and p(C/I) = 1*
**Socio-economic status**					
Low	197 (41)	84 (35)	180 (37.5)	182 (37.9)	*p(I/LE) = 0.04*
Medium	180 (37.5)	102 (42.5)	80 (33.3)	233 (48.5)	*p(C/RS;I) = 0.03/0.001*
High	103 (21.5)	54 (22.5)	47 (19.6)	65 (13.5)	*p (RS;LE/C) = 0.007/0.01*
**Membership of a local saving association**					
No	450 (93.8)	135 (56.3)	190 (79.5)	423 (88.1)	*p (RS/LE;I;C) = < 0.05 and p(C/LE;I) = < 0.05*
Yes	30 (6.3)	105 (43.8)	49 (20.5)	57 (11.9)	*p (LE/RS;I;C) = < 0.001 and p(I/RS;C) = < 0.05*

Data are n (%) and median (interquartile range)

The bar (/) means that the conditions for applying the test are not met

For continuous variables, all *p*-values of the Kruskall-Wallis test were < 0.001. Therefore, we presented in the table the *p*-value not significant for the tests two by two (Wilcoxson test with Bonferroni correction). For the categorical variables, all the proportions were different on the Chi-square test except for sex. We represent in the table the p value of the Z comparison test of column proportions with Bonferroni correction. Eg: p (RS / LE) = 0.01 means that the proportion of people “Never married” in accessible stable health zones is statistically higher than the proportion of people “Never married” in remote and stable health zones; but that it is identical to the proportion of people “Never married” in intermediate health zones and in crisis

### WHODAS score by health zone types

**Fig. 1:**
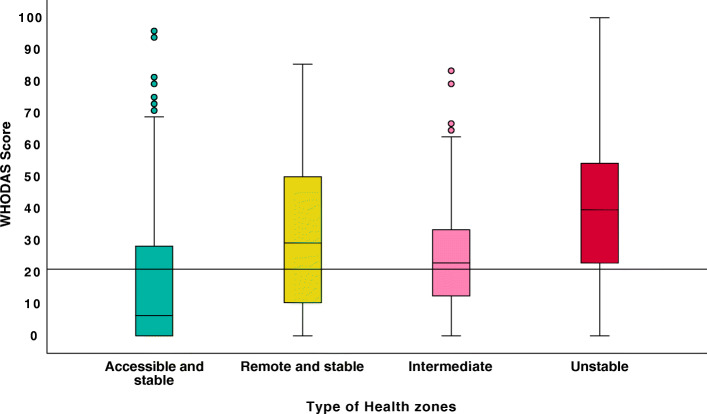
WHODAS score distribution by health zone types

Distributions of WHODAS score by types of health are represented in Fig. 1. The median (Interquartile range) [med (IQR)] overall score of the participants was 25.0 (6.3–41.7). Only the score obtained by the IHZ and RS HZ were not statistically different. Table 2 provide comparaison of the WHODAS score in the Health Zones types. The UHZs had the highest score. Comparing the median scores of the health zones according to the six domains of disability, we notice that: (1) the IHZ has at least one similar item with each of the three other types of health zones; (2) The stable health zones (accessible and remote) have markedly different scores from those of the UHZ on almost all items; (3) there were no statistically significant difference between the median score of the AS and IHZs concerning participation in the life of society (society); so as for the IHZ and UHZ considering taking care of oneself (Self-care). The same applies to the RS and IHZ regarding cognitive aspects and interaction with other members of the community (Cognitive and Social).

**Table 2:** Comparison of the WHODAS score in the health zone types (*n* = 1440)

Items**	Accessible and stable (AS)	Remote and stable (RS)	Intermediate (I)	Unstable (U)	p*
Household	0 (0–4)	3 (1–5)	2 (1–4)	4 (2–6)	< 0.001
Cognitive	0 (0–3)	**2 (0–5)**	**2 (1–3)**	3 (2–5)	< 0.001
Mobility	0 (0–3.75)	3 (0–6)	2 (1–3)	4 (2–6)	< 0.001
Self care	0 (0–0)	0 (0–2)	**1 (0–2)**	**1 (0–3)**	< 0.001
Social	0 (0–0)	**0 (0–2)**	**1 (0–2)**	1 (0–4)	< 0.001
Society	**1 (0–3)**	3 (1–5)	**1.5 (0–3)**	4 (2–5)	< 0.001
WHODAS score	6.3 (0,28.6)	**25 (6.3,41.7)**	**22.9 (12.5,33.3)**	39.6 (22.9,54.2)	< 0.001

Median (IQR).

*p-value of Kruskal-Wallis test

**Items: 1. Assist and complete daily tasks (2 and 12 = Household); 2. Acquisition and use of information (3 and 6 = cognitive); 3. Move around and manipulate objects (1 and 7 = Mobility); 4. Take care of yourself (8 and 9 = Self-care); 5. Interact with others (10 and 11 = Social); 6. Participation in society (4 and 5 = Society)

On each line, cells with numbers in bold indicate where the p-value of the Wilcoxson test with Bonferroni correction is > 0.05

This figure shows that only the accessible and stable health zone has a median score below the severe disability cut-point of 21. The crisis health zone had the highest median WHODAS score. It should also be noted that even in the stable accessible and intermediate zones where the median WHODAS scores are lowest, there are still some people with relatively severe disability.

### Factors associated with high WHODAS score

Table 3. summarizes the results of the hierarchical linear regression model assessing the independent associations between socio-demographic factors and the type of health zone with the WHODAS score. We chose the second model which had the lower AIC.

**Table 3:** Results of the hierarchic multiple linear regression analysis of WHODAS score (*n* = 1440)

Variables	Model 1			Model 2		
	Unstandardized coefficients B	Standardized coefficients B	p	Unstandardized coefficients B	Standardized coefficients B	p
Age	0.377	0.290	< 0.001	0.356	0.274	**< 0.001**
Sex	4.109	0.091	0.001	5.776	0.129	**< 0.001**
Marital statues	2.053	0.074	0.01	2.147	0.077	**0.003**
Respondent’s occupation	−2.447	− 0.137	< 0.001	−1.334	−0.075	**0.002**
Membership of an association	0.939	0.068	0.006	5.944	0.100	**< 0.001**
Number of children < 5 years old in the household	0.975	0.070	0.004	0.224	0.016	0.485
Type of housing	−2.235	−0.071	0.006	−1.838	− 0.059	**0.014**
Number of adults in the household	−0.910	−0.085	0.001	−0.348	− 0.033	0.163
Socio-economic status	0.191	0.006	0.803	0.415	0.014	0.595
Level of crisis of the HZ	–	–	–	6.780	0.387	**< 0.001**
R^2^	0.177			0.312		
F	33.497			63.422		
Significance of the model	< 0.001			< 0.001		
Variation of R^2^	0.177			0.135		
F of the variation of R^2^	33.497			273.927		
Significance of the variation of R^2^	< 0.001			< 0.001		
AIC^a^	8475.396			8225.278		

Coding information: Sexe: 0 = Male, 1 = Female; marital status: 0 = never married, 1 = married, 2 = separated or divorced, 3 = widowed; Respondent’s occupation: 0 = formal employee, 1 = part-time employee, 2 = small trader, 3 = cultivator, 4 = unemployed; Member of saving organization: 0 = No, 1 = Yes; Housing: 0 = temporary, 1 = semi-permanent, 2 = permanent; Socioeconomic status: 0 = low, 1 = medium, 2 = elevate; Level of crisis: 0 = reachable stable, 1 = reachable landlocked, 2 = Intermediate, 3 = crisis;^a^ *AIC* Akaike information criteria

In this model, the factors significantly associated with an overall poor health status (or higher WHODAS score) were; advanced age (B = 0.356; *p* < 0.001), being woman (B = 5.776; *p* < 0.001), being membership of an association (B = 5.944; *p* < 0.001), being divorced, separated or widower (B = 2.147; *p* = 0.003) and living in an unstable health zone (B = 6.780; *p* < 0.001).

Factors significantly associated with an overall higher health status (or lower WHODAS score) were having a permanent housing (B = − 1.838; *p* = 0.014) and having no formal employment (− 1.334; *p* = 0.002).

## Discussion

Our study found that the overall WHODAS score in our population was high, mostly in unstable HZs [Med (IQR) = 39.6 (22.9–54.2)] indicating an overall low health status for the population in this area. The RA and IHZs had globally identical scores and more precisely on the items concerning the cognitive and social aspects. The socio-cultural and demographic characteristics of the participants as well as the type of health zone were associated with the WHODAS score, explaining respectively 17.7 and 13.5% of its variance. The factors significantly associated with an overall poor health status (or higher whodas score) were; advanced age, being woman, being membership of an association, being divorced, separated or widower and living in an unstable health zones.

### The health status of the population in crisis situations related to the armed conflicts

The median WHODAS score obtained in our general population was 25.0(6.3–41.7). Compared to other studies in Africa, the median WHODAS score in the general population was almost the same for people with good cognitive performance [25.0 (IQR 9.0–41.7)] [42]. From this threshold, these high median WHODAS scores were observed in people with mild cognitive impairment [42], or a chronic disease such as HIV [43].

In our study, the median WHODAS score in the unstable health zone was 39.6, which was significantly higher than in the stable health zones (6.3 and 25.0) or even intermediate (22.9). The median score of UHZ would then be found in the 10% of the class with severely impaired disability [40]. Even if there is no consensus on the cut-point defining people with an altered health condition from the WHODAS score [40], our results confirm that the populations living in UHZ have a more impaired health status than those living in stable areas.

We note that poor health status of the population living in unstable health zone in South Kivu concerns all six areas of disability, more particularly the cognitive aspects [Med (IQR) = 3 [2–5]], the execution of daily tasks [Med (IQR) = 4 [2–6]], mobility [Med (IQR) = 4 [2–6]] and participation in the social life of the community [Med (IQR) = 4 [2–5]]. Indeed, armed conflicts create a climate of insecurity and impact on the socio-economic and psychological daily lives of people [11, 44]. WHODAS 2.0 has proven to be an effective tool for assessing disability caused by post-traumatic stress disorder [45]. The chronic crisis related to armed conflicts can be a stressful situation which can lead to disability associated with post-traumatic stress disorder symptoms. The poor health status regarding cognitive aspects can be justified by the fact that armed conflicts may lead to mental disorders [46–48]. For example, in a meta-analysis by Steel et al. the prevalence of posttraumatic stress disorder and depression among people who have experienced torture and other traumatic events was, respectively, 30.6% (95% CI, 26.3–35.2%) and 30.8% (95% CI, 26.3–35.6%) [49]. People live in fear of being attacked again and no longer go about their daily tasks. Unfortunately, most of them, as our study shows (58%), live on small trade of their local products, which is not a stable source of income. The destruction of infrastructure (as well as health structures), the theft of property and physical assault affect emotionally and destroys the community life of the victims.

Our study also showed that aspects related to social life [Med (IQR) = 1 (0–4)] and self-care [Med (IQR) = 1 (0–3)] were the least affected in a crisis setting. This could be explained by the fact that in crisis settings people are more likely to help each other in order to ensure their survival. Furthermore, the crisis also leads to frequent displacement of populations, pushing people to live in temporary housing, as shown in our results. This nomadic life will expose the population to communicable and rapidly fatal diseases mainly due to the lack of drinking water and a very poor environmental sanitation [50, 51].

The remote and stable health zone had an overall WHODAS score statistically identical to that of the intermediate health zone. This suggests that isolation (related to lack of communication and transport infrastructures) during armed conflict could in itself be a factor that can influence the health status of the population. Indeed, it has been noted that populations living in rural and isolated regions are vulnerable in terms of health [52]. Vulnerability, which may increase during armed conflicts in neighboring regions, is mainly due to the shortage of healthcare infrastructure and qualified healthcare personnel in these regions [53].

### Factors associated with variance in WHODAS score

Our second hierarchical regression model showed that socio-cultural and demographic factors account for 19% of the variance in the WHODAS score in our population. Similarly to findings from other studies [40, 54], older age (B = 0.356; *p* < 0.001) and female sex (B = 5.776; *p* < 0.001) seem to be associated with poorer health status. Indeed, it is especially the health status of vulnerable people (women, children, elderly people) which assigned during armed conflicts [55–57]. This may be linked to the fact these vulnerable people find it more difficult to adapt to nomadic life, marked by epidemic diseases, created by displacement during armed conflict [58, 59].

We also note that the WHODAS score decreases in people living in more comfortable dwellings (B = − 1.838; *p* = 0.014). Indeed, having a sustainable and permanent habitat would be a fact which can protect people from environmental and psycho-social risks. It is often people who have not moved in armed conflict who may have these types of accommodation, while IDPs often stay in camps with poorly sanitized permanent accommodation.

Our results also show that the individual’s health status improves if he or she does not have a formal job (B = − 1.334; *p* = 0.002), which is rather curious. Nevertheless, in situations of armed conflict, since it is the psycho-social aspects that are affected, the unemployed may be favored. Indeed, they may have a lot of time to take care of themselves, be more present in their community and may be less stressed by the demands of work.

Our results also suggest that being separated/divorced/widower (B = 2.147; *p* = 0.003) and being a member of an association (B = 5.944; *p* < 0.001) were associated with higher whodas score. These factors can be decisive in the sense that a person’s state of health also depends on his relationship with others [60], further alters the socio-economic dynamics of the population living in these conflict zones.

On the other hand, being a member of an association should rather help to better support the crisis situation. Nevertheless, this could be explained by the fact that during periods of conflict, the created associations are dissolved, leading to a setback in the economic life of the person.

This model also showed that the unstable health zone (crisis areas) explains the variance in the WHODAS score of the population living there at 13.5%. People living in “crisis” health zones had higher WHODAS scores (B = 6780; *p* < 0.001). These results are in line with those from others studies [4, 57, 61, 62] which have found that armed conflicts have a negative impact on the state of health of the population and in several other areas of daily life.

### Strenghts and limitations

Some limitations of our work are worth discussing. Firstly, concerning the selection of health areas and participants: three health areas initially chosen at random were not visited due to accessibility and insecurity issues. However, they were replaced by three other HA contiguous to the previous ones, better accessible and more secure areas. Also, it was more likely that people who went to work could not be found when visiting homes. Thus, we have implemented a double pass system as outlined in the methodology. Second, the fact that the WHODAS tool was not translated into local language (Kibembe, Kitembo, Kirega, Mashi) ​​may affect understanding of the questions. We ensured a good translation of the tool in French and Swahili by a language school according to the principle of translation and counter-transduction advocated by WHO and we pre-tested it. Also, we chose doctors as investigators, guided locally by a community leader. It is difficult to generalize these results to the entire population (note that we used only 6 on 34 health zones of South Kivu), especially since each community lives in a very specific and very often complex state of crisis. The state of health in this case is the result of several other individual, socio-cultural and environmental parameters which are difficult to grasp. However, our study shows that living in a crisis health zones is an important factor which contribute to the poor health status of the population. Finally, for IDPs we used data from the territories to re-calibrate them to the health zones because of the unavailability of disaggregated data by health zones on the OCHA website despite multiple requests. Nevertheless, armed conflicts resulting in IDPs usually take over a whole territory. Or the consequences in terms of population displacement are often spread over the whole territory and not just the area concerned.

Our study nevertheless presents some strengths. It is among the first to study the state of health of the general population in areas affected by crises related to armed conflicts in South Kivu. This is particular, especially since in most cases, the health status of the population is assessed through disease-based or heath programs indicators for the management of these diseases. WHODAS allowed us to see the state of the population’s health from a different perspective, linked to development capacity. This could guide policy makers to have a another view of the real health status of the population, especially those in regions of chronic crisis.

## Conclusions

The crisis related to armed conflicts is a factor which impacts on persons’ health status in several dimensions of their daily life. The assessment of health status of the population in this situation must consider the daily life of the person. The measures usually used, such as those of morbidity and mortality, do not allow a good understanding of the state of health of the person under these conditions. The WHODAS score turns out to be a more suitable to assess the health status of populations living in humanitarian contexts. Health system research and humanitarian actions should focus on the population in health zones undergoing chronic crisis related to armed conflicts to adapt actions according to the aspects of daily life that are altered by these conflicts. WHODAS would then be an essential tool to establish this health status in a comprehensive way (specially people’s psychosocial well-being).

## Data Availability

The datasets used and/or analyzed during the current study are available from the corresponding author on reasonable request.

## Abbreviations

AIC: Akaike information criteria
ASHZ: Accessible and stable Health Zone
BRD: Battle-related Deaths
DRC: Democratic Republic of Congo
HZ: Health Zone
IDPs: Internally Displaced Persons
IHZ: Intermediate Health Zone
Med (IQR): Median (Interquartile Range)
QGIS: Quantum Geographic Information System
RSHZ: Remote and Stable Health Zone
UCDP BRD: Uppsala Conflict Data Program Battle-related Deaths Dataset
UHZ: Unstable Health Zone
UNOCHA: United Nations Office for the Coordination of Humanitarian Affairs
WHODAS: World Health Organization Disability Assessment Schedule
